# Editorial: Utilization of pharmacogenomics in clinical practice

**DOI:** 10.3389/fgene.2024.1470698

**Published:** 2024-08-20

**Authors:** Apostolos Papachristos, Jessica Cusato, Sujit Nair, Simran Maggo, Vijayaprakash Suppiah

**Affiliations:** ^1^ Oncology R&D, AstraZeneca, Waltham, MA, United States; ^2^ Department of Medical Sciences, University of Turin, Turin, Italy; ^3^ Phytoveda Pvt. Ltd., Mumbai, India; ^4^ Bernard J. Dunn School of Pharmacy, Shenandoah University, Virginia, United States; ^5^ Clinical and Health Sciences, University of South Australia, Adelaide, Australia; ^6^ Australian Centre for Precision Health, University of South Australia, Adelaide, Australia

**Keywords:** pharmacogenomic (PGx) research, clinical practice, genomic education, pharmacology, adverse drug (event), drug efficacy and safety

Pharmacogenomics (PGx), the study of how an individual’s genetic makeup affects their response to medication, is increasingly gaining importance in clinical practice. By analyzing patients’ genetic profiles, healthcare providers can make more precise and personalized decisions related to medication selection, dosing, and monitoring. This editorial highlights the potential benefits of utilizing PGx to tailor drug interventions, emphasizing the significance of adopting these advancements in clinical practice to enhance patient care.

The application of PGx with tailored treatments presents the next step forward in precision psychiatry. In this Research Topic, there are three articles that have investigated the use of PGx in psychiatry. In the first study, De Pieri et al. have investigated the clinical utility of polygenic risk score (PRS) to predict response to antipsychotic treatment in a real-world cohort. The authors showed that the PRS was significantly associated in predicting response in all patients, in those diagnosed with schizophrenia, schizoaffective disorder and bipolar. When considered as single diagnosis, only schizophrenia was significantly associated with an OR of 1.27. However, the level of specificity and sensitivity for these associations were below 67% which limits the utility of the PRS in clinical settings.

In their narrative review, Roberts et al. have provided a comprehensive overview of the current knowledge and application of PGx in youth with mental illness. Apart from CYP enzymes, in particular CYP2D6 and CYP2C19, variants from other genes have also been studied in cohorts investigating their association to response time, remission rates, therapeutic concentrations and adverse drug reactions. The implementation of PGx in youth mental health is not without its challenges. Lack of clinical guidelines for youth, attitudes and prior experience of clinicians in implementing PGx testing, patient knowledge and attitudes towards PGx testing and societal expectations are just some of the hurdles that impede the implementation of PGx testing in this vulnerable cohort.

Healthcare professionals need to be equipped with the knowledge to implement PGx testing as well as the tools to integrate the results into clinical decision making. This requires ongoing professional development programs for professionals already in the workforce and incorporation of PGx in the current healthcare curricula. In the third study, Kaltenrieder et al. aimed to measure the changes in knowledge and perceptions of psychiatric mental health nurse practitioner students before and after the implementation of an educational intervention. The authors showed a significant improvement in students’ knowledge and skills related to PGx as well as students’ perceived ability to implement PGx testing in their clinical environment. This study has highlighted the importance of upskilling all healthcare professionals, especially as clinical practice is increasingly becoming multi-disciplinary and prescribing done by other allied healthcare professionals.

In this Research Topic, two papers have described the genetic variability of the N-acetyltransferase 2 (*NAT2*) gene, a key player in drug metabolism, in Brazilian populations and the challenges and solutions for clinical implementations of PGx in Quebec, Canada.

Human NAT2 activity, influenced by several polymorphisms, has been extensively studied and shown to vary significantly among different ethnic groups. The Brazilian study, with its robust sample size of 964 individuals representing five distinct geographical regions, delved into the genetic variability of NAT2 gene in the Brazilian population. Investigators identified a total of 23 previously described NAT2 single nucleotide polymorphisms (SNPs) by sequencing the entire coding exon of NAT2. The main allelic groups included NAT2 5 (36%), NAT2 4 (20.4%), NAT2 6 (18.2%), and when combined into genotypes, the most prevalent were NAT2 5/5 (14.6%), NAT2 5/6 (11.9%), and NAT2 6/*6 (6.2%). Findings revealed significant variability in the *NAT2* slow acetylation phenotype between different geographical regions. Notably, the acetylation profile was found to be homogeneous among the North and Northeast but significantly different from the Southeast (*p* = 0.0396), which was significantly different from all other regions (*p* < 0.0001).

Li et al. described the barriers preventing clinical implementation of PGx in Quebec (Canada). Authors proposed a clinical implementation model adapted to the province’s community, resources, and perspectives. Quebec’s healthcare system is a public system following a single-payer, publicly funded model, meaning that all residents have access to universal coverage. Cost, lack of guidelines, ethical issues, insurance, and the lack of educational resources for clinicians were highlighted as the main barriers associated with PGx implementation as perceived by Quebec healthcare professionals and patients. The utilization of pre-emptive, panel-based PGx and NGS technologies were proposed to improve cost-effectiveness. Moreover, the authors suggested adopting clinical guidelines published by international organizations and the proactive involvement of governmental agencies to produce references. In terms of ethical and insurance considerations, laws such as the Genetic Non-Discrimination Acts of Canada were in place, preventing discrimination based on genetic information. Finally, several Canadian resources and initiatives from local Quebec universities could improve the knowledge by educating healthcare professionals. Thus, the authors concluded that Quebec possesses the essential resources to advance the implementation of PGx testing within their clinical settings.

Wung et al. presented a review of delayed hypersensitivity reactions (DHRs), specifically SCARs associated with antibiotics and anti-osteoporotic drugs (AODs). The authors reported strong associations with specific genetic ancestry, as reported previously. For example, (i) **Co-trimoxazole:** Strongly linked with *HLA-B13:01* (OR: 45; *p*-value: 1.1 × 10^−26^) for DRESS in Chinese, Thai, and Malaysian populations. *HLA-B15:02* was associated with SJS/TEN (OR: 2.7 to 3.91; *p*-values: 0.008 to 0.0037); (ii) **Penicillins:** *HLA-B55:01* was associated with penicillin allergy in Europeans (OR: 1.30; *p*-value: 10–47), while *HLA-DRB302:02* was linked to delayed hypersensitivity in Italians (OR: 8.9; *p*-value: <0.001); (iii) **dapsone:** HLA-B*13:01 was linked to hypersensitivity in Chinese, Thai, and Indonesian populations (OR: 49.64 to 328.87; *p*-values: 2.92 × 10^−4^ to 1.32 × 10^−7^); (iv) **Vancomycin:** HLA-A*32:01 was strongly associated with DRESS in Europeans, Spanish, and Chinese (OR: 403; *p*-values: 10-8 to 0.035); (v) **Strontium Ranelate:** HLA-A*33:03 was linked to SJS/TEN in Chinese populations (OR: 19.4; *p*-value: 0.006). These associations have highlighted the importance of pre-emptive PGx testing in patients of predisposed ancestry to prevent these severe adverse drug reactions.

Additionally, a Korean study investigated the relationship between CoQ10-related gene polymorphisms and statin-related myotoxicity (SRM) in 688 stroke patients of whom 56 had experienced SRM. The authors reported that certain SNPs related to CoQ10 biosynthesis were significantly associated with the risk of SRM. Specifically, the *COQ2* rs4693075*G allele, *COQ3* rs11548336*TT genotype, and *COQ5* rs10849757*A allele were associated with a higher risk of SRM. It was found that atorvastatin decreased the risk of SRM, whereas ezetimibe use increased it.

Manson et al. investigated the feasibility identifying specific HLA alleles that increased the risk of adverse drug reactions and explored the potential impact of using this data in clinical practice. The study concluded that repurposing HLA genotype data from kidney transplant patients was feasible and can significantly contribute in preventing drug hypersensitivity reactions. In the cohort of 1,347 kidney transplant recipients, 13.1% carried at least one of the four HLA risk alleles (*HLA-A31:01, HLA-B*15:02, *HLA-B57:01,* and *HLA-B58:01*). These patients were at increased risk for hypersensitivity reactions to drugs such as flucloxacillin and allopurinol. Although no hypersensitivity reactions were observed during the study, the integration of HLA genotype data into electronic health records and the subsequent generation of PGx alerts helped healthcare providers make tailored medication decisions.

Also included in this Research Topic is a methods paper for rapid and high-resolution HLA class I typing using transposase-based nanopore sequencing. Authors reported transposase-based nanopore sequencing method with the Oxford Nanopore Rapid Barcoding kit, reducing hands-on time from 9 h to 4 h and lowering costs compared to traditional ligation-based sequencing methods. This novel approach was tested on 20 DNA samples, including 11 from diverse ethnicities and nine from Thai individuals, using two primer sets for gene amplification. Both commercial and in-house bioinformatics tools were employed for the data analysis. Results showed that transposase-based sequencing achieved over 90% accuracy, comparable to the GeT-RM standard. The method provided high-resolution HLA typing, identifying key alleles associated with drug hypersensitivity, such as HLA-B*15:02, with high accuracy and speed. Cost analysis revealed that transposase-based sequencing reduced the cost per sample to approximately $270, compared to $335 for ligation-based sequencing, making it a more affordable option for routine clinical use. This cost-effectiveness, combined with its rapid turnaround positions transposase-based nanopore sequencing as a promising tool for enhancing PGx testing and preventing adverse drug reactions, particularly in resource-limited settings.

In conclusion, the contribution of PGx in clinical practice has the potential to revolutionize how healthcare providers prescribe drugs, leading to more tailored and effective treatment plans for patients. As advancements in PGx continue to expand, it is important for healthcare providers to be upskilled and integrate this valuable tool into their clinical practice for improving patient care and for all stakeholders to be appropriately involved.

